# Meta-Analysis of the Reasoned Action Approach (RAA) to Understanding Health Behaviors

**DOI:** 10.1007/s12160-016-9798-4

**Published:** 2016-05-11

**Authors:** Rosemary McEachan, Natalie Taylor, Reema Harrison, Rebecca Lawton, Peter Gardner, Mark Conner

**Affiliations:** Bradford Institute for Health Research, Bradford Teaching Hopsitals NHS Foundation Trust, Bradford, BD9 6RJ UK; School of Psychology, University of Leeds, Leeds, LS2 9JT UK; Australian Institute of Health Innovation, 75 Talavera Road, Macquarie University, North Ryde, NSW 2109 Australia

**Keywords:** Reasoned action approach, Theory of planned behavior, Meta-analysis, Health behavior, Protection behaviors, Risk behaviors

## Abstract

**Background:**

Reasoned action approach (RAA) includes subcomponents of attitude (experiential/instrumental), perceived norm (injunctive/descriptive), and perceived behavioral control (capacity/autonomy) to predict intention and behavior.

**Purpose:**

To provide a meta-analysis of the RAA for health behaviors focusing on comparing the pairs of RAA subcomponents and differences between health protection and health-risk behaviors.

**Methods:**

The present research reports a meta-analysis of correlational tests of RAA subcomponents, examination of moderators, and combined effects of subcomponents on intention and behavior. Regressions were used to predict intention and behavior based on data from studies measuring all variables.

**Results:**

Capacity and experiential attitude had large, and other constructs had small-medium-sized correlations with intention; all constructs except autonomy were significant independent predictors of intention in regressions. Intention, capacity, and experiential attitude had medium-large, and other constructs had small-medium-sized correlations with behavior; intention, capacity, experiential attitude, and descriptive norm were significant independent predictors of behavior in regressions.

**Conclusions:**

The RAA subcomponents have utility in predicting and understanding health behaviors.

Identifying the factors predicting engagement in health behaviors has been the focus of considerable research in health psychology. A variety of social cognition models purporting to delineate the key determinants of behavior [1] have been applied to health behaviors. These prominently include the theory of planned behavior (TPB; [2]), an extension of the theory of reasoned action (TRA; [3]). The TPB is a parsimonious model applied to a wide range of health behaviors (for reviews, see [4–10]). In recent years, researchers have sought to develop the TPB by differentiating subcomponents of the model [11, 12]. Despite including very similar constructs, this expanded model (Fig. 1) has been given a variety of names (e.g., two-factor model; [12]). We here refer to this model as the reasoned action approach (RAA; [13]). The present paper provides a meta-analytic review of the RAA subcomponents to health behaviors, test of health behavior type (protection vs. risk) as a moderator, and test of the power of the subcomponents to independently predict intention and behavior.

**Fig. 1:**
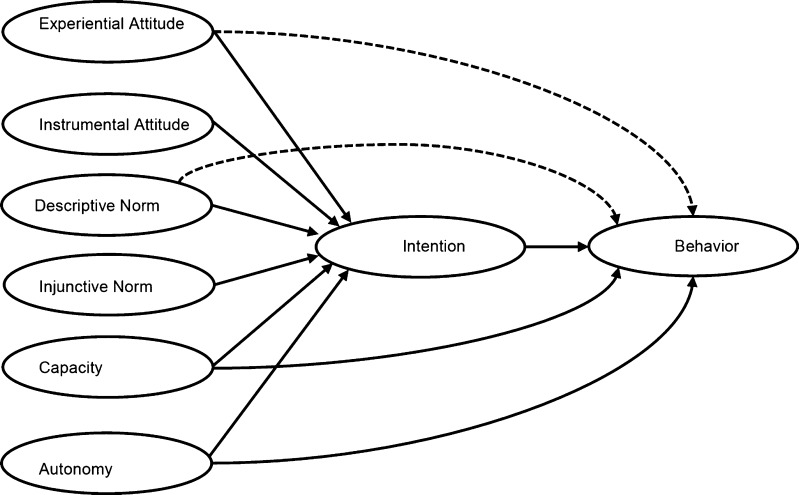
The subcomponent RAA (*dashed lines* indicate additional paths suggested by the meta-analysis)

## Overview of the TPB and RAA

The TPB states that behavior is determined by an individual’s behavioral intention and that perceived behavioral control (PBC) also determines behavior [2] or moderates the impact of intention on behavior [13]. Intention is held to be the motivational component that spurs an individual to engage in a particular behavior. PBC captures the extent to which people have control over engaging in the behavior or confidence that they can perform the behavior. Intention, in turn, is determined by an individual’s attitude toward the behavior (e.g., whether engaging in the behavior is evaluated to be positive or negative), subjective norms (e.g., perceptions of whether others think one should engage in a behavior), and PBC. Overall, the TPB has been shown to explain 40–49 % of the variance in intention and 26–36 % of the variance in behavior [2, 5, 7, 8, 14, 15]. The most comprehensive meta-analysis of prospective health behavior TPB studies to date [10] reported that intention and PBC explained 19.3 % variance in behavior, while attitude, subjective norm, and PBC explained 44.3 % variance in intention. This meta-analysis also reported that the predictive ability of the TPB varied across different health behaviors.

In the RAA (Fig. 1), the three determinants of intentions are labeled attitude toward the behavior, perceived norm, and PBC [13], with each represented by pairs of distinct, but related, subcomponents [11–13, 16]. In particular, attitude toward the behavior is assumed to consist of experiential and instrumental attitudes; perceived norm is assumed to consist of injunctive and descriptive norms, while PBC is assumed to consist of capacity and autonomy ([13]; see below for definitions). Ajzen and Fishbein [11] have suggested that the subcomponents reflect the more general construct (e.g., experiential and instrumental attitudes reflect overall attitude toward the behavior) and that the more general constructs be used in analyses (i.e., a second-order factor analysis model). Although this has the advantage of parsimony, it has the disadvantage of requiring further theorizing about the relationship between the more general construct and the subcomponents [12]. Considering each of the subcomponents as independent predictors of intention and behavior is the approach taken in the growing number of studies reviewed here. This approach has the advantage of allowing us to examine which subcomponent is the more important predictor and also to test novel pathways (e.g., experiential attitude to behavior). This, in turn, allows us to more precisely specify targets of intervention to change health behaviors. Previous meta-analyses of the TPB have explored subcomponents of the RAA in isolation (e.g., experiential vs. instrumental attitudes [17]; injunctive vs. descriptive norms [18]; capacity vs. autonomy [19]) and pointed to the discriminant validity of the subcomponents. However, there is no meta-analysis of the power of all six subcomponents as zero-order and as independent predictors of intention and behavior. The present meta-analysis addresses this gap in relation to health behaviors and explores type of behavior (i.e., health protection and health-risk behaviors) as a moderator.

The semantic differential measures of attitude toward behavior used in TPB studies often focus more on instrumental or cognitive (e.g., healthy–unhealthy, valuable–worthless) compared to experiential or affective (e.g., pleasant–unpleasant, interesting–boring) aspects of attitude [20, 21]. Studies have found experiential measures of attitude to be more closely linked to intentions [20, 22–25] and behavior [25, 26]. In the RAA, separate measures tap experiential/affective and instrumental/cognitive components of attitude. The two components have medium-sized correlations with one-another [17] but can be discriminated based on their underlying belief systems [27], different functions [28], experimental manipulations [29], and empirical differences [30]. It is suggested that instrumental attitude may impact behavior through a “reflective” path via intention, while experiential attitude operates both via intention and through an “impulsive” direct path to behavior ([25, 31]; Fig. 1).

The perceived norm component of the TPB has also been a focus of research, with the unexpectedly weak predictive power of subjective norm being noted by some authors [5, 32, 33]. One explanation for this weak predictive power is the focus on injunctive norm [34]. Cialdini et al. [34] label the norms in the TPB injunctive norm as they concern the perceived social approval of others which motivates behavior through social reward/punishment, and distinguish them from descriptive norm which are perceptions of what others do [35]. Although some authors argue that injunctive and descriptive norms be considered indicators of the same underlying concept [3, 13, 36], recent research has pointed to their discriminant validity. In a meta-analysis, Rivis and Sheeren [37] found that descriptive norm explained an additional 5 % of variance in intention after taking account of attitude, injunctive norm, and PBC. Similarly, Manning [18] reported that in a meta-analysis of TPB studies measuring injunctive and descriptive norms, injunctive norm was a stronger correlate of intention compared to descriptive norm (*r*_*+*_ = 0.51, *k* = 160; *r*_*+*_ = 0.40, *k* = 17), although the pattern was reversed for predictions of behavior (*r*_*+*_ = 0.28, *k* = 156; *r*_*+*_ = 0.34, *k* = 17). The two constructs show medium-large-sized correlations with one another ([37], *r*_*+*_ = 0.38; [18], *r*_*+*_ = 0.59) [38]. In distinguishing injunctive and descriptive norms, the RAA allows testing of their independent effects on intention and behavior. In so doing, it helps identify distinct pathways of effect on behavior (e.g., injunctive norm may influence behavior only indirectly through intention as hypothesized in the TPB, while descriptive norm might indirectly influence behavior through intentions and also directly influence behavior reflecting modeling or other processes; Fig. 1).

The difference between the TPB and the earlier TRA lies in the addition of PBC. Meta-analytic reviews support the power of PBC to explain additional variance in intention and behavior after controlling for the components of the TRA [5]. However, overlaps between PBC and self-efficacy [39] have long been noted (see also [13]). Some researchers have advocated the use of measures of self-efficacy in place of PBC alongside components of the TRA [40]. Opinion appears to have coalesced around the idea of PBC tapping two separate but related constructs [13, 15, 19, 41], although the preferred terminology varies. We follow Fishbein and Ajzen [13] in labeling these constructs as capacity and autonomy. Capacity “…deals with the ease or difficulty of performing a behavior, with people’s confidence that they can perform it if they want to do so” [42]. Capacity shows considerable overlap with many definitions of self-efficacy (sometimes also labeled capability). Autonomy “… involves people’s beliefs that they have control over the behavior, that performance or non-performance of the behavior is up to them” [42]. Armitage and Conner [5] reported that capacity (that they labeled self-efficacy) compared to autonomy (labeled perceived control) were stronger correlates of intention (*r*_*+*_ = 0.44 vs. 0.23) and behavior (*r*_*+*_ = 0.35 vs. 0.18). The RAA, in distinguishing these two components, allows tests of their independent effects on intention and behavior and highlights different pathways of effect on behavior (e.g., capacity influencing behavior both directly and indirectly through intention, while autonomy only directly influencing behavior independent of intention).

The TRA/TPB explicitly states that the power of different components to predict different behaviors might vary [13]. Indeed, one meta-analysis [10] reported type of behavior to be a key moderator of model relationships. Consistent differences between clusters of health behaviors could be expected on theoretical grounds and if confirmed might help guide intervention efforts. For example, the prototype-willingness model (PWM; [43]) is an adaption of the TRA/TPB specifically for risk behaviors in adolescent groups that particularly emphasizes the role of normative influences. Similarly, Conner et al. [17] emphasized the role of experiential/affective influences in applications of the TPB to risk behaviors. The present research reports a meta-analysis of published RAA studies focusing on comparing the pairs of subcomponents, testing differences between health protection versus health-risk behaviors (e.g., previous research suggests that experiential attitudes [17] or descriptive norms [43] may be stronger predictors of behavior for risk compared to protection behaviors), and testing differences in the power of the subcomponents of the RAA to predict intention and behavior in regressions based on those studies measuring all variables.

## Method

### Searches

Relevant databases (PsycINFO, MEDLINE, Web of Science, CINAHL, Embase) were searched on two occasions (10 May 2010 and 6 November 2012), using the following search strings: (1) attitud* and norm* and control and intention*; OR (2) theory of planned behavi*; OR (3) planned behavi* and Ajzen. Citation searches were performed on three key papers [2, 5, 7], and content pages of key journals were searched (British Journal of Health Psychology, Health Education Research, Health Psychology, Journal of Applied Social Psychology, Psychology and Health). Key authors were contacted to identify additional eligible articles not otherwise identified.

### Selection Criteria

The inclusion/exclusion criteria for studies were based on those of McEachan et al. [10], with the addition of one criterion related to measurement of the RAA:

1. Prospective study providing measure of behavior at follow-up.
2. Measuring health behavior. Health behaviors were defined as behaviors which impact or have the potential to impact on the health of an individual in a positive or negative way and included behaviors such as physical activity, safer sex, drug use, and screening.
3. Explicitly testing the TPB and providing overall measures of attitude toward behavior, perceived behavioral control, intention, and either overall perceived norm (*k* = 35) or a belief-based measure of norm (*k* = 7).
4. Reporting a minimum sample size of *N* = 30, reporting zero-order correlations between at least one pair of subcomponent variables and intention or behavior.
5. Measuring at least instrumental *and* experiential attitude, or injunctive *and* descriptive norm, or capacity *and* autonomy.

Following McEachan et al. [10], we excluded studies if they reported cross-sectional or retrospective assessment of behavior; if they described interventions different from “normal care” where no control analyses were reported; and if they reported studies involving professional athletes, descriptions of physician behavior, patient “help-seeking” behavior, or dieting/weight control among general population samples. Studies providing only a “stage of change” algorithm measure of behavior were also excluded. Papers from meeting abstracts, theses, or other unpublished research were not included.

**Fig. 2:**
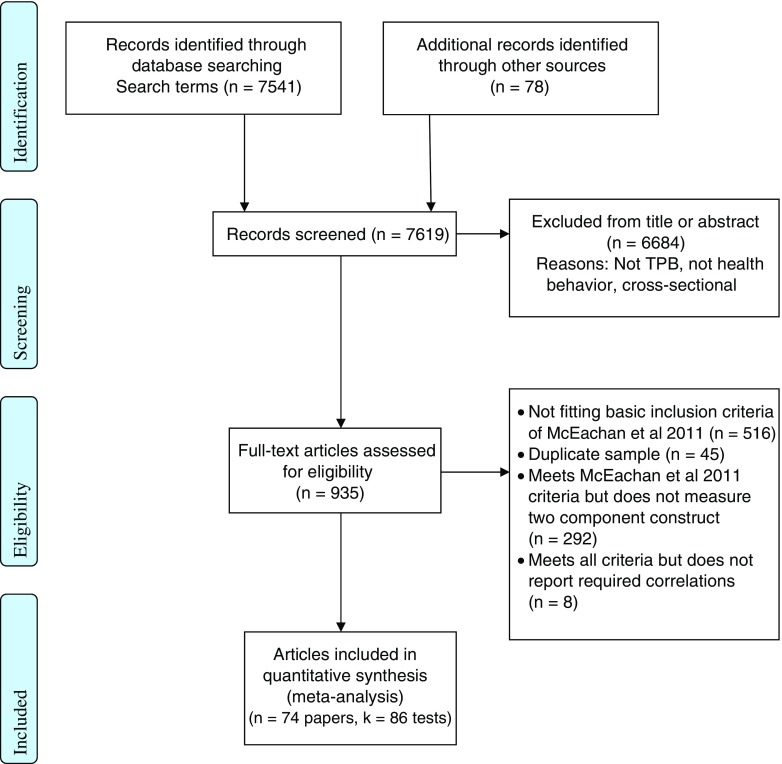
Flow of studies for meta-analysis

A total of 7619 citations were identified. The first author performed an initial screen and excluded 6684 on the basis of their abstract or title. The full text of the remaining 935 articles were downloaded and then reviewed in a two-stage process. First, articles were screened according to inclusion criteria 1 to 4: 561 were excluded. The full text of the remaining 374 were scrutinized to ascertain whether the paper reported instrumental and experiential attitude, or injunctive and descriptive norm, or capacity and autonomy. A further 292 were excluded. A randomly selected 10 % of articles were independently screened by a second reviewer for inclusion and exclusion, agreement was 96 %; disagreements were discussed and resolved. A total of 82 papers were found to be eligible (Fig. 2), of these 74 papers (including 86 tests) provided sufficient information to be included in the review. A total of 62 (76 %) of the 82 papers were included in the McEachan et al.’s [10] meta-analysis.

### Data Abstraction

In order to identify which subcomponent RAA variables studies assessed, all items used to measure RAA constructs were extracted from included studies. A code book was developed and piloted (using [12, 42]) to assign measures as assessing general, subcomponent, or belief-based measures of TPB variables (Table 1). One reviewer coded all measures while a second blind coded approximately 50 % (average kappa = 0.97). Disagreements were resolved by discussion.

**Table 1:** List of studies included in meta-analysis (study 1; *k* = 86)

	RAA measures reported			Study characteristics			
Study	Experiential and instrumental attitudes	Injunctive and descriptive norm	Capacity and autonomy	Sample size (*N*)	Type of behavior	Sample type	Follow-up (weeks)
Abraham, Henderson, and Der (2004) [44]		✓		260^a^ 2048^b^	Protection: using condoms	Adolescent/school: school children	Longer, 130
Armitage and Conner (1999a) [45]			✓	94	Protection: eating a low fat diet	Student	Shorter, 4
Armitage and Conner (1999b) [46]			✓	413	Protection: eating a low-fat diet	Adult: workforce	Longer, 13
Armitage, Conner, Loach, and Willetts (1999) [47]			✓	121	Risk: multiple drug behaviors, drink alcohol, smoke cannabis	Student	Shorter, 2
Berg, Jonsson, and Conner (2000) [48]		✓		1086^a^ 1500^b^	Protection: healthy eating	Adolescent/school: school children	Shorter, 3
Bish, Sutton, and Golombok (2000) [49]		✓	✓	133	Detective: health screening	Adult: general public	Longer, 13
Blanchard et al. (2009a) [50]	✓			511	Protection: eating five fruit and vegetables	Student	Shorter, 1
Blanchard et al. (2009b) [51] Sample 1: Caucasian Sample 2: African American	✓ ✓			176 237	Protection: eating five fruit and vegetables Protection: eating five fruit and vegetables	Student Student	Shorter, 2 Shorter, 2
Blanchard et al. (2008a) [52] Sample 1: Caucasian Sample 2: African American	✓ ✓			197 238	Protection: physical activity Protection: physical activity	Student Student	Shorter, 1 Shorter, 1
Blanchard et al. (2008b) [53] Sample 1: Caucasian Sample 2: African American	✓ ✓			273 280	Protection: physical activity Protection: physical activity	Student Student	Longer, 8 Longer, 8
Boudreau, Godin, Pineau, and Bradet (1995) [54]		✓		86	Protection: physical activity	Adult: workforce	Longer, 8
Bryan and Rocheleau (2002) [55]		✓		204	Protection: physical activity (aerobic and resistance exercise)	Student	Longer, 13
Chatzisarantis, Hagger, Wang, and Thogersen-Ntoumani (2009) [56]		✓		231	Protection: physical activity	Adolescent/school: school children	Longer, 5
Conner, Godin, Sheeran, and Germain (2012) [57]	✓	✓		1070	Other: donating blood	Adult: general public	Longer, 26
Conner and McMillan (1999) [58]		✓		118	Risk: using illegal drugs	Student	Longer, 13
Conner, Rhodes, Morris, McEachan, and Lawton (2011) [29] Study 1: Control group Study 2: Control group	✓ ✓	✓ ✓	✓ ✓	61 32	Protection: physical activity Protection: physical activity	Student Student	Shorter, 3 Shorter, 3
Conner, Rodgers, and Murray (2007) [59]	✓	✓		146	Protection: physical activity	Student	Shorter, 1
Conner, Sherlock, and Orbell (1998), Study 2 [60]		✓	✓	123	Risk: using illegal drugs	Adult: general public	Longer, 8
de Bruijn, Keer, van den Putte, and Neijens (2012) [61]	✓			109	Protection: fruit consumption	Student	Shorter, 4
de Bruijn, Rhodes, and van Osch (2012) [62]	✓			415	Protection: physical activity	Student	Shorter, 3
de Bruijn, Verkooijen, de Vries, and van den Putte (2012) [63]	✓			413	Protection: physical activity	Student	Shorter, 2
de Vries, Backbier, Kok, and Dijkstra (1995) [40]		✓		401	Risk: smoking	Adolescent/school: school children	Longer, 26
Dunn, Mohr, Wilson, and Wittert (2011) [64]		✓	✓	401	Risk: fast food	Adult: general public	Shorter, 1
Elliott and Ainsworth (2012) [65]	✓	✓	✓	120	Risk: binge drinking	Student	Shorter, 2
Elliott and Thomson (2010) [66]	✓	✓	✓	1403	Risk: speeding	Adult: general public	Longer, 26
Ellis Gardner and Hausenblas (2004) [67]	✓			58^c^	Protection: exercising and eating a healthy diet	Adult: overweight women	Shorter, 4
Eves, Hoppe, and McLaren (2003) [68]	✓			133^a^ 233^b^	Protection: six separate physical activity behaviors (e.g., aerobics, swimming)	Adult: general public	Shorter, 4
Giles, Liddell, and Bydawell (2005) [69]			✓	32	Protection: using condoms	Adolescent/school: young people	Shorter, 1
Giles, McClenahan, Cairns, and Mallet (2004) [70]			✓	55^a^ 100^b^	Other: donating blood	Student	Shorter, 1
Godin, Anderson, Lambert, and Desharnais (2005) [71]		✓		740	Protection: physical activity	Adolescent/school: school children	Longer, 52
Hagger and Chatzisarantis (2005) [72] Study 1 Study 2	✓ ✓	✓ ✓	✓ ✓	523 596	Protection: dieting Protection: exercise	Student Adult: workforce	Shorter, 2 Shorter, 2
Hoie, Moan, Rise, and Larsen (2012) [73] Sample 1: Young people Sample 2: General Public	✓ ✓	✓ ✓		174 272	Other: Quitting smoking Other: Quitting smoking	Adolescent/school: young people Adult: general public	Longer, 13 Longer, 13
Jackson, Smith, and Conner (2003) [74]		✓	✓	85	Protection: physical activity	Adult: workforce	Longer, 8
Karvinen et al. (2009) [75]	✓	✓		397	Protection: physical activity	Adult: patient	Longer, 12
Kellar and Abraham (2005) [76] control group			✓	69	Protection: eating five fruit and vegetables	Student	Shorter, 1
Kraft, Rise, Sutton, and Roysamb (2005) [77]	✓		✓	110	Protection: physical activity	Student	Shorter, 2
Lawton, Ashley, Dawson, Waiblinger, and Conner (2012) [78] Sample 1: White British Sample 2: South Asian	✓ ✓	✓ ✓	✓ ✓	71 113	Other: breastfeeding Other: breastfeeding	Adult: pregnant mothers Adult: pregnant mothers	Longer, 26 Longer, 26
Lawton, Conner, and McEachan (2009) [25]	✓	✓	✓	390	Multiple Risk (binge drinking, alcohol, illegal drugs, smoking exceeding speed limit) Protection (brushing teeth, exercising, flossing, fruit and vegetable consumption, low fat diet, physical activity, self-examination, sunscreen use, vitamin use)	General public	Shorter, 4
Lowe, Eves, and Carroll (2002) [79]	✓			365	Protection: physical activity	Adult: general public	Longer, 26
McEachan, Sutton, and Myers (2010) [80]	✓	✓	✓	397^a^ 418^b^	Protection: physical activity	Student	Shorter, 2
McMillan and Conner (2003) [81]		✓		139	Risk: using illegal drugs	Student	Longer, 26
McMillan et al. (2008) [82]		✓		248	Other: breastfeeding	Adult: general public	Longer, 6
McMillan, Higgins, and Conner (2005) [83]		✓		620^a^ 741^b^	Other: not initiating smoking	Adolescent/school: school children	Longer, 13
Molla, Astrom, and Berhane (2007) [84]		✓		743	Protection: using condoms	Adult: general public	Longer, 12
Myers and Horswill (2006) [85]			✓	46	Protection: using sunscreen	Student	Longer, 16
Norman (2011) [86]			✓	109^a^ 137^b^	Risk: binge drinking	Student	Shorter, 4
Norman, Armitage, and Quigley (2007) [87]			✓	79^a^ 94^b^	Risk: binge drinking	Student	Shorter, 1
Norman and Conner (2006) [88]			✓	273^a^ 398^b^	Risk: binge drinking	Student	Shorter, 1
Norman and Hoyle (2004) [89]			✓	95	Detective: breast self-examination	Adult: workforce	Shorter, 4
Payne, Jones, and Harris (2002) [90]			✓	199	Protection: physical activity	Adult: workforce	Shorter, 1
Payne, Jones, and Harris (2004) [91]	✓			296	Protection: physical activity and healthy eating	Adult: workforce	Shorter, 1
Plotnikoff, Courneya, Trinh, Karunamuni, and Sigal (2008) [92]		✓		244	Protection: physical activity	Adult: patient	Longer, 12
Povey, Conner, Sparks, James, and Shepherd (2000a) [93]		✓		234^a^ 242^b^	Protection: healthy eating	Adult: general public	Shorter, 2
Povey, Conner, Sparks, James, and Shepherd (2000b) [94] Sample 1 Sample 2			✓ ✓	143 144	Protection: eating a low-fat diet Protection: eating five fruit and vegetables	Adult: general public Adult: general public	Shorter, 4 Shorter, 4
Raudsepp, Viira, and Hannus (2010) [95]	✓			236	Protection: physical activity	Adolescent/school: school children	Longer, 52
Rhodes and Blanchard (2008) [96], sample 2	✓			174	Protection: physical activity	Student	Shorter, 2
Rhodes, Blanchard, Matheson, and Coble (2006) [97]	✓			230	Protection: physical activity	Student	Shorter, 2
Rhodes and Courneya (2003) [98]	✓			305	Protection: physical activity	Student	Shorter, 4
Rhodes and Courneya (2005) [99]	✓			585	Protection: physical activity	Student	Shorter, 2
Rhodes, Courneya, Blanchard, and Plotnikoff (2007) [100]	✓			358	Protection: physical activity	Adult: general public	Longer, 8
Rhodes, de Bruijn, and Matheson (2010a) [101]	✓			153	Protection: physical activity	Student	Shorter, 2
Rhodes, Jones, and Courneya (2002) [102]	✓			192	Protection: physical activity	Student	Shorter, 2
Rhodes and Matheson (2005) [103]	✓			241	Protection: physical activity	Student	Shorter, 2
Rhodes, Matheson, and Mark (2010) [104] Sample 1 Sample 2 Sample 3 Sample 4	✓ ✓ ✓ ✓	✓ ✓ ✓ ✓	✓ ✓ ✓ ✓	111 100 99 101	Protection: physical activity Protection: physical activity Protection: physical activity Protection: physical activity	Student Student Student Student	Shorter, 2 Shorter, 2 Shorter, 2 Shorter, 2
Rise, Kovac, Kraft, and Moan (2008) [105]	✓	✓		103	Other: quitting smoking	Student	Longer, 16
Rivis and Sheeran (2003b) [106]		✓		225^a^ 333^b^	Protection: physical activity	Student	Shorter: 2
Rodgers, Conner, and Murray (2008) [19], study 1			✓	278	Protection: physical activity	Student	Shorter, 1
Schutz et al. (2011) [107] Scott, Eves, French, and Hoppe (2007) [108], study 2	✓	✓		237 130^a^ 139^b^	Protection: using condoms Protection: physical activity	Adult: males Adult: army trainees	Longer, 26 Shorter, 1
Scott et al. (2010) [109]	✓			186	Protection: physical activity	Adult: army trainees	Shorter, 1
Shankar, Conner, and Bodansky (2007) [110]	✓		✓	54	Detective: checking blood glucose levels	Adult: patient	Shorter, 2
Sieverding, Matterne, and Ciccarello (2010) [111]		✓		2307	Detective: health screening	Adult: general public	Longer, 52
Victoir, Eertmans, Van den Bergh, and Van den Broucke (2005) [112]	✓			80	Protection: safe driving	Adult: general public	Shorter, 1
Wilkinson and Abraham (2004) [113]	✓	✓		225	Risk: smoking	Adolescent/school: school children	Longer, 26
Woolfson and Maguire (2010) [114]			✓	62	Risk: binge drinking	Student	Shorter, 4
Number of tests (*k*)^d^	49^e^	42^f^	36				

^a^Sample size for relationships with behavior

^b^Sample size for relationships with intention

^c^Pooled sample size (69 for exercise, 46 for diet)

^d^Three studies failed to report intention–behavior correlation [40, 107, 111] resulting in *k* = 83 tests

^e^Two studies failed to report correlation of experiential or instrumental attitude with behavior [68] or intention and behavior [113]

^f^Two studies failed to report correlation of descriptive norm with behavior [84] or descriptive norm and injunctive norm with behavior [111]

### Moderator Coding

Studies that assessed at least one pair of RAA subcomponents were blind double coded for further characteristics by two reviewers. All disagreements were discussed and resolved. In line with previous reviews [10], we coded the type of health behavior in the study, the type of sample, and time delay from measurement of RAA variables to measurement of behavior (Table 1). It was not possible to include coding of other moderators used in previous meta-analyses of the TPB (e.g., objective vs. self-report measures of behavior) because of limited numbers of studies in at least one category.

#### Type of health behavior

Following Rothman and Salovey [115], we coded studies into protection (approach), risk (avoidance), or other (e.g., detection, curative) health behaviors. Fifty-nine tests were coded within the protection category. This category included physical activity behaviors (*k* = 41), behaviors related to a healthy diet (*k* = 14), using condoms (*k* = 4) or sunscreen (*k* = 1), and safe driving (*k* = 1). Risk behaviors included drinking alcohol, smoking, using drugs, or exceeding the posted speed limit (*k* = 15). Other behaviors (*k* = 12) included detection behaviors (general health screening, *k* = 2; breast self-examination, *k* = 1; checking blood glucose levels among type 1 diabetic patients, *k* = 1), quitting smoking (*k* = 3), breastfeeding (*k* = 3), and blood donation (*k* = 2). Due to their heterogeneity, other behaviors were not included in the moderator analyses of behavior type. One paper [25] reported both risk and protection behaviors assessed from one sample. The appropriate correlations from this study were included in estimates for both types of behavior but the sample size halved. Agreement for coding of behavior was 97 %, and disagreements were resolved through discussion.

#### Type of sample

Type of sample was coded into adolescent or school age (e.g., those recruited directly from schools or youth clubs and who were 17 years or younger, *k* = 10), student (e.g., undergraduate or postgraduate student samples recruited from university settings, *k* = 41), or adult samples (e.g., excluding latter two groups and recruited from community settings, *k* = 30) (cp. [10]). One study contained a mixture of age groups, and four could not be coded (e.g., recruited military trainees; [109]) and so excluded from analyses on type of sample. Agreement for coding was 89 % with disagreements resolved by discussion.

#### Time interval between measures

This was coded in weeks but was highly skewed (median 3.5 weeks; range = 1–130 weeks). Studies were therefore split into two groups labeled shorter follow-up (≤4 weeks, *k* = 54) or longer follow-up (>4 weeks, *k* = 32). Coding agreement was 99 % with disagreements resolved by discussion.

### Analysis

Analyses were conducted using Comprehensive Meta-Analysis software, version 2.2.064 [116], and SPSS, version 20, using correlation coefficients extracted from papers. A random effects meta-analysis was performed, with effect size estimates weighted by sample size. Mean effect sizes (*r*_*+*_), standard deviations, heterogeneity estimates (*Q* statistic; [117]), percentage of variation accounted for by statistical artifacts (*I*^2^), and fail-safe numbers (FSN) were computed. Significant *Q* values were indicative of significant heterogeneity. The *I*^2^ statistic was used to quantify the degree of heterogeneity (*I*^2^ values of 25, 50, and 75 % indicate low, moderate, and high levels of heterogeneity, respectively; [118]). FSNs were compared against tolerance levels to assess potential file drawer problems [119]. We also used Egger’s regression test [120] and the Duval and Tweedie’s [121] trim and fill procedure to identify potential publication bias. Moderator analyses of our categorical moderators were undertaken using random effects subgroup analyses. Moderator analyses were only conducted where every category contained at least three studies. Variance between studies was expected to be consistent across subgroups, and thus, the heterogeneity variance within each subgroup (*τ*^2^) was estimated by a single value collapsing across subgroups [116]. Statistical significance of each moderator was assessed using *Q* tests analogous to analysis of variance, such that a significant between-group *Q* indicates that the effect size differs significantly as a function of the moderator. Proportion of heterogeneity accounted for by each moderator was computed using adjusted *R*^2^ (ratio of variance explained by the moderator relative to the amount of variance in total), calculated using 1 − (*τ*^2^ within / *τ*^2^ total) [116].

In order to explore the simultaneous impact of predictor variables in explaining intention and in explaining behavior, we used multiple regression based on the frequency-weighted mean correlations. However, given the fact that the number of tests contributing to estimates of individual correlations varied so much, leading to potential problems of nonpositive definite matrices, we decided to base these analyses only on those studies (*k* = 14, *N* = 3990) that included *all* components of the RAA. We report the correlation matrix that regressions were based on. For regressions predicting intention, instrumental and experiential attitudes, injunctive and descriptive norms, capacity and autonomy were entered simultaneously. For regressions predicting behavior, at a first step, we entered intention, capacity and autonomy (to parallel the TPB), followed by all remaining variables at step 2. For each step of each regression, we report the percentage additional variance explained (Δ*R*^2^) and the independent contribution of each construct in the form of unstandardized beta weights (B), standard errors (SE), and standardized beta weights (β).

## Results

### Overview of Tests

Table 1 shows that of the total pool of 86 tests, 49 tests compared instrumental and experiential attitudes (of these, *k* = 39 were coded as preventive, *k* = 3 as risk, *k* = 7 as other), 42 tests compared injunctive and descriptive norms (*k* = 23 preventive, *k* = 9 risk, *k* = 10 as other), and 36 tests compared autonomy and capacity (*k* = 20 preventive, *k* = 10 risk, *k* = 6 as other). There were only 14 full tests of the full subcomponent model from eight papers. Table 1 also shows the coding of each study by type of sample and time interval between measures.

### Overall Effect Sizes

Table 2 shows the meta-analysis correlations for each of the six constructs with intention and behavior; intercorrelations between the pairs of constructs are also reported. *Z* tests were used to compare the relative magnitude of subcomponent constructs correlations with intention and behavior. Capacity and experiential attitude showed large-sized correlations with intention, while instrumental attitude, injunctive norm, descriptive norm, and autonomy showed medium-large-sized correlations with intention. The intercorrelations between instrumental and experiential attitudes, injunctive and descriptive norms, and capacity and autonomy were of medium-large magnitude (Table 2; [38]). Intention, capacity, and experiential attitude all showed medium-large-sized correlations with behavior. Instrumental attitude, descriptive norm, autonomy, and injunctive norm showed small-medium-sized correlations with behavior. Compared to instrumental attitude, experiential attitude showed significantly stronger associations with both intention (experiential: *r*_*+*_ = 0.546; instrumental: *r*_*+*_ = 0.384) and behavior (experiential: *r*_*+*_ = 0.299; instrumental: *r*_*+*_ = 0.195). The same pattern was apparent for capacity versus autonomy (intention: capacity: *r*_*+*_ = 0.598; autonomy: *r*_*+*_ = 0.268; behavior: capacity: *r*_*+*_ = 0.388; autonomy: *r*_*+*_ = 0.189). There were also significant but more modest differences in the magnitude of correlations between injunctive or descriptive norms with intention (injunctive norm: *r*_*+*_ = 0.389; descriptive norm: *r*_*+*_ = 0.351) or behavior (injunctive norm: *r*_*+*_ = 0.220; descriptive norm: *r*_*+*_ = 0.265).

**Table 2:** Meta-analysis correlation estimates for behavior and intention

	*N*	*k*	*r*_+_	95 % CI	*Q* value^a^	*df*	*I* ^2^	FSN	Diff
Intention–behavior	21,245	83	0.481	0.441–.518	1074.39	82	92.37	106,267	–
Experiential attitude–behavior	12,724	47	0.299	0.260–.338	248.15	46	81.46	12,938	11.75*
Instrumental attitude–behavior	12,724	47	0.195	0.145–.244	363.21	46	87.34	5950	–
Injunctive norm–behavior	12,191	40^b^	0.220	0.181–.259	177.85	39	78.07	56,549	4.67*
Descriptive norm–behavior	12,191	40	0.265	0.220–.310	255.92	39	84.76	7945	–
Autonomy–behavior	7109	36^c^	0.189	0.139–.237	138.60	35	74.75	2232	17.35*
Capacity–behavior	7109	36	0.388	0.338–.435	174.68	35	79.96	9544	–
Experiential attitude–intention	13,019	48	0.546	0.503–.586	510.40	47	90.79	50,074	21.01*
Instrumental attitude–intention	13,019	48	0.384	0.332–.434	528.63	47	91.11	24,049	–
Injunctive norm–intention	18,110	42	0.389	0.348–.428	373.14	41	89.01	26,392	5.10*
Descriptive norm–intention	18,091	42	0.351	0.315–.387	274.01	41	85.04	21,458	–
Autonomy–intention	7424	36^c^	0.268	0.197–.336	331.34	35	89.44	4820	32.24*
Capacity–intention	7424	36	0.598	0.550–.643	307.47	35	88.62	26,728	–
Experiential attitude–instrumental attitude	12,389	46	0.457	0.414–.498	368.56	45	87.79	31,854	–
Injunctive norm–descriptive norm	18,091	42	0.386	0.328–.440	754.95	41	93.91	25,894	–
Autonomy–capacity	7424	36^c^	0.427	0.340–.507	640.80	35	94.54	2735	–

*N* total number of participants in included test, *k* total number of studies, *r*_*+*_ frequency-weighted correlation, *95 % CI* 95 % confidence interval around *r*_*+*_, *df* degrees of freedom for *Q* value, *FSN* fail safe number, *I* ^*2*^ *I*-squared, *Diff Z* test of difference between magnitude of subcomponent variable correlation with intention or behavior

^a^All values of *Q* statistic *p* < 0.05; *p < 0.05

^b^ *k* = 41, *r*_+_ = 0.220 (95 % CI = 0.182–0.258) when including one additional study [84] reporting the descriptive norm-behavior but not the injunctive norm-behavior correlation

^c^These values include correlations from five studies [65, 86–88, 114] on binge drinking where the negative correlations were reversed to be consistent with other studies. Excluding these five studies gave the following values: autonomy–behavior: *k* = 31, *r*_+_ = 0.195 (95 % CI = 0.141–0.248); autonomy–intention: *k* = 31, *r*_+_ = 0.286 (95 % CI = 0.209–0.359); autonomy–capacity: *k* = 31, *r*_+_ = 0.473 (95 % CI = 0.389–0.550)

Egger’s regression test revealed significant asymmetry (*t*s > 1.86, *ps* < 0.05) for six correlations (four correlations with behavior and two with intention). For each correlation, we then used the Trim and Fill method [121] to examine effects sizes after studies were trimmed compared to those reported in Table 2. In one case, the values reduced by 0.003 (capacity–behavior: one study trimmed; *r*_*+*_ = 0.385, 95 % CI 0.335–0.432), while in the other cases, the values increased by between 0.006 and 0.048 (instrumental attitude–behavior: eight studies trimmed; *r*_*+*_ = 0.232, 95 % CI 0.185–0.278; injunctive norm–behavior: nine studies trimmed; *r*_*+*_ = 0.266, 95 % CI 0.227–0.305; autonomy–behavior: nine studies trimmed; *r*_*+*_ = 0.232, 95 % CI 0.187–0.277; instrumental attitude–intention: nine studies trimmed; *r*_*+*_ = 0.414, 95 % CI 0.355–0.470; autonomy–intention: eight studies trimmed; *r*_*+*_ = 0.331, 95 % CI 0.266–0.393). The fail safe number (FSN) for effects reported in Table 2 ranged from a low of 1848 (for autonomy–behavior) to a high of 106,267 (for intention–behavior). These findings suggest the influence of publication bias in the meta-analysis can be designated as modest rather than severe.

The above results appear unlikely to be unduly influenced by the reliability of the measures of each construct. The majority of studies used multiple items to measure each construct (ranging from 71 % of studies for autonomy to 100 % of studies for experiential attitude) and the reliability of these multi-item scales was generally good (mean Cronbach’s alphas ranging from 0.72 for descriptive norms to 0.82 for experiential attitudes). Excluding studies with low reliabilities (alpha < 0.60) did not substantively alter the Table 2 correlations.

### Moderator Analyses

In relation to the correlations reported in Table 2, all *Q* values were significant (*ps* < 0.001) and the *I*^2^ statistic ranged between 74.75 and 94.54 %, indicating moderate to high levels of heterogeneity for all correlations. We assessed the impact of type of behavior and sample as moderators of all correlations and time interval between measures as a moderator of relationships with prospective measures of behavior (Table 3). In relation to type of behavior, there were significant moderation effects for eight relationships. Intention, experiential attitude, instrumental attitude, injunctive norm, and descriptive norm were each significantly stronger correlates of behavior in risk compared to protection behaviors. In addition, experiential attitude and instrumental attitude were significantly stronger correlates of intention in risk compared to protection behaviors. Finally, the autonomy–capacity correlation was significantly stronger in protection compared to risk behaviors (Table 3).

**Table 3:** Moderator analyses

	Moderator	*Q value*	*df*	Subsample	*k*	*r*_+_	95 % CI	*I* ^2^	% Het
Intention–behavior	Type of behavior^a^	6.13^*^	1	Protection	59	0.479	0.434–0.522	91.9	10.40
				Risk	13	0.599	0.515–0.672	86.2	
	Type of sample	1.74	2	School	9	0.443	0.322–0.551	87.8	2.20
				Students	41	0.516	0.464–0.565	87.9	
				Older	28	0.479	0.413–0.540	94.3	
	Length of follow-up	6.45^*^	1	Shorter	53	0.517	0.471–0.561	90.9	4.20
				Longer	30	0.414	0.345–0.479	93.1	
Experiential attitude–behavior	Type of behavior^a^	10.03^*^	1	Protection	38	0.304	0.262–0.345	76.3	0.00
				Risk	3	0.525	0.399–0.630	89.9	
	Type of sample	0.00	1	School	2	–			
				Students	28	0.306	0.254–0.357	79.6	0.00
				Older	14	0.308	0.234–0.378	68.2	
	Length of follow-up	3.44	1	Shorter	34	0.323	0.277–0.368	84.7	6.00
				Longer	13	0.242	0.166–0.315	84.7	
Instrumental attitude–behavior	Type of behavior^a^	10.09^*^	1	Protection	38	0.186	0.132–0.239	85.9	16.70
				Risk	3	0.475	0.311–0.612	25.2	
	Type of sample	0.81	1	School	2	–			
				Students	28	0.183	0.117–0.247	87.3	4.00
				Older	14	0.234	0.144–0.321	88.3	
	Length of follow-up	0.02	1	Shorter	34	0.197	0.136–0.256	86.7	0.00
				Longer	13	0.188	0.093–0.280	89.5	
Injunctive norm–behavior	Type of behavior^a^	3.97^*^	1	Protection	23	0.211	0.162–0.258	70.5	18.00
				Risk	9	0.299	0.226–0.369	73.5	
	Type of sample	4.46	2	School	7	0.269	0.184–0.351	76.5	0.00
				Students	15	0.261	0.193–0.327	54.0	
				Older	17	0.179	0.118–0.238	84.8	
	Length of follow-up	2.21	1	Shorter	16	0.258	0.194–0.319	70.8	0.00
				Longer	24	0.196	0.145–0.247	81.2	
Descriptive norm–behavior	Type of behavior^a^	8.15^*^	1	Protection	23	0.258	0.208–0.306	74.0	26.70
				Risk	9	0.386	0.313–0.454	76.2	
	Type of sample	11.93^*^	2	School	7	0.393	0.301–0.478	91.7	10.50
				Students	15	0.291	0.214–0.363	54.2	
				Older	17	0.193	0.124–0.261	85.7	
	Length of follow-up	0.09	1	Shorter	16	0.274	0.199–0.346	66.5	0.00
				Longer	24	0.260	0.200–0.318	88.9	
Autonomy–behavior	Type of behavior^a^	0.59	1	Protection	21	0.208	0.141–0.272	80.6	0.00
				Risk	10	0.165	0.071–0.255	56.3	
	Type of sample	0.74	1	School	0	–			
				Students	19	0.164	0.095–0.231	50.5	6.30
				Older	15	0.207	0.131–0.278	83.9	
	Length of follow-up	0.02	1	Shorter	28	0.190	0.131–0.247	78.4	0.00
				Longer	8	0.181	0.072–0.286	46.2	
Capacity–behavior	Type of behavior^a^	0.05	1	Protection	21	0.400	0.335–0.461	76.1	0.00
				Risk	10	0.412	0.321–0.494	84.5	
	Type of sample	0.42	1	School	1	–			
				Students	19	0.380	0.312–0.444	66.2	9.10
				Older	16	0.411	0.343–0.475	84.5	
	Length of follow-up	0.83	1	Shorter	29	0.403	0.346–0.457	69.8	0.00
				Longer	8	0.346	0.233–0.451	91.2	
Experiential attitude–intention	Type of behavior^a^	4.12*	1	Protection	39	0.536	0.487–0.582	89.6	0.00
				Risk	3	0.694	0.549–0.799	96.0	
	Type of sample	1.80	1	School	2	–			
				Students	28	0.571	0.514–0.622	89.6	0.00
				Older	15	0.505	0.420–0.582	93.2	
Instrumental attitude–intention	Type of behavior	5.89*	1	Protection	39	0.373	0.311–0.431	91.4	4.40
	Type of sample	0.89	1	School	2	–			
				Students	28	0.282	0.316–0.446	91.4	5.30
				Older	15	0.435	0.347–0.515	87.6	
Injunctive norm–intention	Type of behavior^a^	0.10	1	Protection	24	0.378	0.319–0.435	86.6	0.00
				Risk	9	0.396	0.301–0.483	92.9	
	Type of sample	0.84	2	School	7	0.366	0.277–0.449	87.8	0.00
				Students	15	0.365	0.295–0.430	79.7	
				Older	18	0.401	0.344–0.455	88.5	
Descriptive norm–intention	Type of behavior^a^	3.22	1	Protection	23	0.346	0.298–0.392	75.6	0.00
				Risk	8	0.425	0.351–0.493	89.1	
	Type of sample	1.34	2	School	7	0.378	0.292–0.459	73.6	7.10
				Students	15	0.362	0.294–0.427	79.2	
				Older	18	0.324	0.265–0.381	89.6	
Autonomy–intention	Type of behavior^a^	1.47	1	Protection	21	0.287	0.195–0.374	91.5	0.00
				Risk	10	0.189	0.054–0.317	72.9	
	Type of sample	0.51	1	School	0	–			
				Students	19	0.235	0.131–0.333	87.3	0.00
				Older	15	0.288	0.178–0.392	92.4	
Capacity–intention	Type of behavior^a^	0.38	1	Protection	20	0.606	0.540–0.665	90.2	0.00
				Risk	10	0.572	0.474–0.656	84.6	
	Type of sample	0.41	1	School	1	–			
				Students	19	0.615	0.548–0.675	90.4	0.00
				Older	15	0.584	0.506–0.653	86.9	
Experiential attitude–instrumental attitude	Type of behavior^a^	1.01	1	Protection	37	0.462	0.409–0.511	89.0	0.00
				Risk	3	0.551	0.375–0.688	86.5	
	Type of sample	0.49	1	School	2	–			
				Students	28	0.469	0.411–0.522	86.8	0.00
				Older	13	0.433	0.345–0.513	91.3	
Injunctive norm–descriptive norm	Type of behavior^a^	0.73	1	Protection	24	0.382	0.292–0.466	89.9	0.00
				Risk	9	0.452	0.312–0.572	98.2	
	Type of sample	2.72	2	School	7	0.277	0.127–0.415	91.7	0.00
				Students	15	0.395	0.294–0.487	85.0	
				Older	18	0.413	0.326–0.493	85.0	
Autonomy–capacity	Type of behavior^a^	14.80^*^	1	Protection	21	0.529	0.443–0.606	93.4	36.80
				Risk	10	0.202	0.044–0.349	86.2	
	Type of sample	0.17	1	School	0	–			
				Students	19	0.449	0.327–0.557	94.6	0.00
				Older	15	0.413	0.272–0.536	95.1	

*df* degrees of freedom for *Q* test, *Subsample* groups compared for significant *Qs*, *k* total number of studies in subsample, *r*_*+*_ frequency-weighted correlation for each subsample, *95 % CI* 95 % confidence interval around *r*_*+*_ for each subsample, *I* ^2^ *I*-squared for each subsample, *%Het* percentage of heterogeneity explained for significant moderators

* *p* < .05

^a^Lawton et al. [25] assessed both risk and preventive behavior and was included in both subsamples with sample size halved

In relation to type of sample, only the relationship between descriptive norm and behavior was significantly moderated (Table 3). Subgroup analysis indicated that the strongest correlation was for school samples, then students and finally older samples. In each case, the comparisons between each pair were significant (*Q*s > 8.27, *p*s < 0.001). Finally, in relation to time interval between measures, only the relationship between intention and behavior was significantly moderated (Table 3). Intention was a significantly stronger predictor of behavior in studies with shorter compared to longer time intervals between measurement of cognitions and behavior.

### Predicting Intention and Behavior

Given the considerable variation in the number of studies contributing to the estimate of each bivariate correlation we decided to base our regression analyses on the subset of studies estimating all relationships. This has the advantage of being more likely to produce a positive definite correlation matrix but the disadvantage of limiting the number of studies included. Table 4 reports the correlation matrix used in these regressions. Examination of the relevant correlations in Tables 2 and 4 indicate generally higher values in Table 4 although the relative magnitude of correlations was similar.

**Table 4:** Matrix of correlations based on studies measuring all variables (*k* = 14; *N* = 3990)

	1.	2.	3.	4.	5.	6.	7.	8.
1. Behavior	–	0.551	0.370	0.158	0.414	0.303	0.261	0.285
2. Intention		–	0.564	0.275	0.675	0.530	0.388	0.316
3. Capacity			–	0.479	0.467	0.323	0.223	0.242
4. Autonomy				–	0.174	0.205	0.168	0.157
5. Experiential attitude					–	0.511	0.318	0.263
6. Instrumental attitude						–	0.379	0.160
7. Injunctive norm							–	0.400
8. Descriptive norm								–

Multiple regression analyses of the correlation matrix of average effect sizes for the studies that estimated all relationships in the RAA (*k* = 14; *N* = 3990) indicated experiential attitude, capacity, instrumental attitude, injunctive norm, and descriptive norm were each significant independent predictors of intention explaining 58.7 % of the variance, *F*(6,3983) = 942.4, *p* < 0.001. Autonomy was not a significant predictor of intention. Experiential attitude and capacity were the strongest predictors of intention (Table 5).

**Table 5:** Regressions of intention or behavior onto RAA variables for studies reporting all relationships

	Predicting intention			Predicting behavior (step 1)			Predicting behavior (step 2)		
Predictors	*B*	*SE*	*β*	*B*	*SE*	*β*	*B*	*SE*	*β*
Intention	–	–	–	0.502	0.016	0.502*	0.435	0.020	0.435*
Capacity	0.273	0.013	0.273*	0.100	0.017	0.100*	0.085	0.018	0.085*
Autonomy	0.008	0.013	0.008	–0.028	0.015	–0.028	–0.032	0.015	–0.032
Experiential attitude	0.400	0.013	0.400*	–	–	–	0.051	0.019	0.051*
Instrumental attitude	0.187	0.012	0.187*	–	–	–	0.001	0.016	0.001
Injunctive norm	0.098	0.012	0.098*	–	–	–	0.017	0.015	0.017
Descriptive norm	0.074	0.011	0.074*	–	–	–	0.111	0.015	0.111*

Predicting intention: *R* ^2^ = 0.587; *F*(6,3983) = 942.4, *p* < 0.001. Predicting behavior: step 1, Δ*R* ^2^ = 0.309; Δ*F*(3,3986) = 595.1, *p* < 0.001. Step 2, Δ*R* ^2^ = 0.014; Δ*F*(4,3982) = 21.2, *p* < 0.001

*p < 0.01

In relation to behavior, multiple regression analyses using the same correlation matrix indicated that intention and capacity, but not autonomy, were significant independent predictors (Table 5, step 1) explaining 30.9 % of the variance, *F*(3,3986) = 595.1, *p* < 0.001. Adding experiential attitude, instrumental attitude, injunctive norm, and descriptive norm explained a further 1.4 % of the variance in behavior (*F*(4,3982) = 21.2, *p* < 0.001), with intention, capacity, experiential attitude, and descriptive norm significant at this step (Table 5, step 2). Autonomy, instrumental attitude, and injunctive norm were not significant predictors of behavior. Intention was the dominant predictor of behavior (Table 5), indeed entering intention alone explained 30.4 % of the variance in behavior, B = 0.551, SE = 0.013, *F*(1,3988) = 1738.6, *p* < 0.001.

## Discussion

This meta-analysis assessed the power of the RAA in relation to prospective tests on health behaviors. The RAA with subcomponents extends the TPB by splitting each of attitude toward behavior, perceived norm, and PBC into two subcomponents [13, 122]. The meta-analysis provided support for the discriminant validity of the subcomponent conceptualization of experiential versus instrumental attitudes, injunctive versus descriptive norms, and capacity versus autonomy (i.e., correlations between pairs of constructs was only of medium-large magnitude and did not approach 1.0; Table 2). Capacity and experiential attitudes showed large-sized correlations with intention, while instrumental attitude, injunctive norm, descriptive norm and autonomy showed medium-large-sized correlations with intention. Intention, capacity, and experiential attitude all showed medium-large-sized correlations with behavior, while instrumental attitude, descriptive norm, autonomy, and injunctive norm showed small-medium-sized correlations. Compared to instrumental attitude, experiential attitude showed significantly stronger associations with both intention and behavior. The same pattern was apparent for capacity versus autonomy, with the former being a significantly stronger correlate of both intention and behavior. The differences in magnitude of correlations between injunctive or descriptive norms with intention or behavior were more modest but still significant, with injunctive norm being the stronger predictor of intention and descriptive norm being the stronger predictor of behavior (Table 2).

Significant heterogeneity in the correlations was only partly explained by our moderators, suggesting the need to treat these findings with some caution. Our key moderating variable of type of behavior significantly influenced the relationship between intention and behavior, between experiential or instrumental attitudes and intention or behavior, between injunctive or descriptive norm and behavior, and between autonomy and capacity. This suggests interesting directions for future research. Given that experiential attitude, instrumental attitude, and descriptive norm were significant stronger predictors of risk compared to protection behaviors, this might suggest the particular importance of these variables in relation to risk behaviors and the potential value of differentially targeting variables when attempting to change risk versus protection behaviors.

There were also significant moderating effects for our other two moderators. Time delay between measurement of intention and behavior significantly moderated this relationship, supporting previous meta-analyses of the TPB [10] in showing stronger correlations over shorter intervals. This is a limiting condition of the TRA/TPB, although intention can still predict over prolonged time intervals [123]. That the descriptive norm–behavior relationship was significantly stronger in adolescent/school aged compared to adult samples is a noteworthy finding (cp. [10]), particularly given the direct effects of descriptive norm on behavior independent of intention and other constructs (see below). In practical terms, interventionists targeting younger age groups might usefully focus on targeting descriptive norm with strategies such as modeling or group performance.

Regression analyses indicated experiential attitudes and capacity to be the strongest predictors of intention, although instrumental attitude, injunctive norm, and descriptive norm were also significant independent predictors of intention explaining a total of 58.7 % of the variance. Only autonomy was not a significant predictor. This is a higher percentage of variance than reported in previous meta-analyses of the TPB [10], although direct comparisons are difficult given differences in the number of predictors. Nevertheless, this is an impressive amount of explained variance and approaches the limits that may be possible given measurement error in measuring each construct. Regression analyses also indicated that intention was the strongest predictors of behavior, but that capacity, experiential attitude, and descriptive norm were also significant, explaining 32.3 % of the variance. Autonomy, instrumental attitude, and injunctive norm were not significant predictors of behavior. Key differences between the TPB and RAA with subcomponents here are the direct effects that experiential attitude and descriptive norm have on behavior in the RAA and the lack of direct effect on intention or behavior for autonomy in the RAA. Nevertheless, in general, the findings broadly support the RAA with intention being the dominant predictor of behavior [13].

A number of important differences emerge between the RAA and TPB. First, although both experiential and instrumental attitudes are significant correlates of intention and behavior, and both are significant simultaneous predictors of intention in regressions, only experiential attitude is a significant predictor of behavior in regressions controlling for RAA variables. Second, injunctive and descriptive norms are significant correlates of intention and behavior, and both are significant simultaneous predictors of intention in regression. However, only descriptive norm is a significant predictor of behavior in regressions controlling for other RAA variables. Third, of the two constructs making up PBC, a differentiated pattern emerges for capacity and autonomy. Both constructs are significant positive correlates of intention and behavior, although capacity has a significantly stronger correlation in each case (Table 2). Capacity was a significant positive predictor of intention when controlling for other RAA constructs, while autonomy was a nonsignificant negative predictor of intention. Capacity was a significant positive predictor of behavior when controlling for intention and autonomy. Capacity remained a significant predictor of behavior controlling for all RAA constructs. Autonomy was not a significant predictor of behavior in the regressions across behaviors. Fourth, protection-risk behavior comparisons indicated significant moderation effects for correlations. These four key findings are now discussed in more detail.

In relation to the first point, the support for the important role of experiential compared to instrumental attitudes as a predictor of both intention and behavior supports a growing body of research in this area including intervention studies [29]. Experiential attitude appears to be a key determinant of intention and behavior [17]. Independent effects for both attitudes on intention suggest the value of targeting both in relation to changing intention as a means to change behavior (i.e., an indirect effect). Also, the additional significant direct effect of experiential attitude on behavior independent of intention suggests that changing experiential attitude compared to instrumental attitude could have stronger impacts on behavior change given the direct and indirect paths. Further research attempting to independently manipulate experiential and instrumental attitudes and observing effects on intention and behavior (and the relative size of direct and indirect paths to behavior) is required. The direct path by which experiential attitude influences behavior may be particularly important as it suggests a nonreasoned path to behavior (i.e., not mediated by intention; Fig. 1). It might be that experiential attitude better reflects impulsive influences on behavior. Some support for this hypothesis comes from the relative strength of experiential attitude compared with instrumental attitude in predicting the more hedonic and impulsive “risk” behaviors. Interventionists should take into account these patterns of prediction when designing interventions to change protection versus risk behaviors. Nevertheless, the relatively small amount of variance explained by experiential attitude over and above intention indicates the importance of targeting intentions.

In relation to the second point, support for both injunctive and descriptive norms as correlates of intention and behavior is a further important finding. Given that the TPB has previously only focused on injunctive norm, this might help explain why norms appeared to have only a relatively modest impact on intention compared to other constructs. Both injunctive and descriptive norms emerge as independent predictors of intention. Direct effects of descriptive norm on behavior independent of intention may reflect modeling processes (Fig. 1). Further research independently manipulating injunctive and descriptive norms and observing effects on intention and behavior (and the relative size of direct/indirect paths to behavior) is required. Although it is again worth noting that intention is the dominant predictor of behavior.

In relation to the third point, the current findings for capacity and autonomy partially support the idea of research focusing on the former [19, 40]. In the context of health behaviors, capacity was a significantly stronger correlate of both intention and behavior and a more consistent independent predictor of intention and behavior when controlling for other RAA constructs. There were no significant effects for autonomy in regressions. A recent review of drinking alcohol noted that autonomy was negatively related to intention and behavior [124]. Further research exploring effects for autonomy on intention and behavior in risk versus protection health behaviors is warranted. However, the current research provides little support for a focus on autonomy. In contrast, the observed effects of capacity support the emphasis on this construct (labeled as self-efficacy) in other research perspectives such as social cognitive theory [125] and protection motivation theory [126]. However, further research that independently manipulates autonomy and capacity and observes effects on intention and behavior might be useful before focusing attention solely on capacity to the exclusion of autonomy. Indeed, recent research [127, 128] has called for more focus on measures of autonomy rather than capacity because the former is a purer measure of perceived capability. Williams and colleagues [128] suggest that capacity measures (labeled self-efficacy) may reflect motivation rather than perceived capability. The strong predictive power of capacity in relation to intention and behavior could be seen as consistent with the latter point. However, the weak effects for autonomy observed here might suggest a limited predictive power for purer measures of perceived capability such as autonomy (see [129] for a useful discussion of the two aspects of perceived behavioral control that the RAA distinguishes).

In relation to the fourth point, a number of significant differences emerged for the application of the RAA to protection versus risk behaviors. The two consistent findings were the significantly stronger effects for experiential attitudes and descriptive norms on behavior for risk compared to protection behaviors, although both constructs were significant predictors of behavior for both types of behaviors. The meta-analysis indicated significant differences in simple correlations. These findings suggest that it might be valuable to target changes in experiential attitude and descriptive norm in order to directly (independent of intention) change health behaviors, and that this may be a particularly potent approach for risk behaviors. Further research that independently manipulates experiential attitude and descriptive norm and observes effects on behavior for protection versus risk behaviors would be valuable in testing these predictions. In identifying differences between protection and risk behaviors, the present research adds further support for the idea that a “one size fits all” approach to developing interventions is undesirable [10]. Future research could usefully test whether interventions that change the key predictors identified here produces different effects for protection versus risk behaviors. The added value of distinguishing between individual protection or risk behaviors in terms of predictors is also an issue for further research.

The present research has a number of strengths including examining a range of health behaviors. There are also a number of weaknesses including a reliance on self-reported behavior measures and failure to examine the effects of controlling for past behavior (see [130] for useful discussion of this issue). An important further weakness is the lack of a direct comparison of the TPB and RAA. In the present data, the RAA explained 58.7 and 32.3 % of the variance in intention and behavior, respectively, considerably higher than the values previously reported for intention (44.3 %) and behavior (19.3 %) [10]. However, simple direct comparisons of the two in terms of amount of variance explained in intention or behavior are difficult to interpret given the larger number of predictors in the RAA compared to the TPB. Nevertheless, the new pathways to behavior identified here for the RAA (Fig. 1), particularly if supported in subsequent research, may be considered an important advantage that offsets the loss in parsimony for the subcomponent version of the RAA over the TPB. Nevertheless, further studies testing the discriminant validity of pairs of constructs (e.g., instrumental vs. experiential attitude) or novel studies showing that the constructs can be independently manipulated would be particularly valuable in more clearly demonstrating the value of the subcomponents of the RAA over the TPB.

In summary, the present paper indicates the potential value of the subcomponents of the RAA in helping us understand the determinants of health behaviors. Although less parsimonious than the TPB, the RAA with subcomponents offers unique insights into the determinants of health behaviors. Experiential attitude, instrumental attitude, injunctive norm, descriptive norm, and capacity emerge as consistent predictors of intention, while intention, capacity, experiential attitude, and descriptive norm emerge as predictors of behavior. Novel direct effects of experiential attitude and descriptive norm on behavior, independent of intention, suggest important unplanned influences on behavior that might form additional targets for interventions designed to change health behaviors (Fig. 1, dashed lines). An important future test of the RAA subcomponents will be the extent to which the unique insights it provides into the determinants of health behaviors are supported in direct experimental tests of manipulations that test specific pathways in the model.
